# Image quality of an investigational imaging panel for use with the imaging beam line cone‐beam CT

**DOI:** 10.1120/jacmp.v13i1.3607

**Published:** 2012-01-05

**Authors:** Chris Beltran

**Affiliations:** ^1^ Department of Radiological Sciences St. Jude Children's Research Hospital Memphis TN 38105 USA

**Keywords:** MV‐CBCT, image quality, imaging beam line

## Abstract

The purpose of this study was to measure and compare the contrast‐to‐noise ratio (CNR) as a function of dose for the cone‐beam CT (CBCT) produced by the imaging beam line (IBL) for the standard and an investigational imaging panel. Two Siemens Artiste linear accelerators were modified at our institution such that the MV‐CBCT would operate under an investigational IBL. The imaging panel from one of the machines was replaced with an investigational imaging panel. After the modification, a set of CBCT for a large and small phantom consisting of eight tissue‐equivalent inserts was acquired for the standard imager and for the investigational imager with and without the standard copper plate. Ten dose settings for each phantom using the IBL in combination with the standard and investigational imaging panel were acquired. The CNR for each tissue‐equivalent insert was calculated. Resolution measurements in line pairs per mm (lp/mm) of the CBCT for the various imaging panel setups were made. In addition, CBCT images of two patients that were imaged with each panel configuration were displayed for a group of physicians and therapists who were asked to identify the best and worst CBCT for each patient. This was used as a qualitative judge of practical image quality. The CNR of the muscle insert for the large phantom with 1.5 cGy at isocenter was 1.3 for the standard imager, 1.5 for the investigational imager with the copper plate, and 1.9 without the plate. Under the same conditions, the CNR of the trabecular bone insert was 5.9, 7.3, and 9.7, respectively. For the small phantom with the same dose to isocenter, the CNR for muscle was 1.7, 2.1, and 3.3, respectively. For the trabecular bone, the CNR was 8.1, 9.6, and 12.1 respectively. The resolution for 1 cGy at isocenter was 0.37 lp/mm for the standard imager, 0.32 and 0.33 for the investigational imager with and without the copper plate. The qualitative test ranked the CBCT of the investigational imager without the copper plate to be the best image, and the standard imager to be the worst. The investigational imaging panel improves image quality as compared to the standard imager for IBL CBCTs. A 1 cGy IBL CBCT, no matter which imager is used, is sufficient for bony anatomy localization. The investigational imager without the copper plate was judged clinically to produce the best IBL CBCT.

PACS numbers: 87.57.Q, 87.57.cj

## I. INTRODUCTION

In recent years, cone‐beam computed tomography (CBCT)^(^ ^1^ ^)^ has become somewhat routine for treatment localization based on anatomical structures or implanted markers.^(^ ^2^ ^–^ ^11^ ^)^ Since CBCT provides a volumetric dataset, it can be directly registered to the planning simulation CT. There are currently three linac‐based commercially available CBCT systems in the market: two kilovoltage (kV) systems — the Varian On‐Board Imaging (OBI) system (Varian Medical Systems, Palo Alto, CA) and the Elekta XVI Synergy system (Elekta, Stockholm, Sweden), and one megavoltage (MV) system — the Siemens MVision system (Siemens AG, Munich, Germany).^(^ ^12^ ^)^ Recently, Faddegon et al.^(^ ^13^ ^,^ ^14^ ^)^ introduced a modification to the Siemens MV‐CBCT, referred to as the imaging beam line (IBL), which allows for improved image quality at a lower radiation dose as compared to the treatment beam line (TBL). The major modifications consisted of replacing the tungsten target with a carbon target, removing the flattening filter, and decreasing the beam energy by 30%.

We previously published results that compared the contrast‐to‐noise ratio (CNR) as a function of dose for the CBCT produced by the IBL and the TBL. In addition, we compare the dose to the target and to various critical structures of pediatric patients for the IBL CBCT.^(^ ^15^ ^)^ In that paper, we noted that the IBL could produce better quality CBCT at lower doses than the TBL. We subsequently reported localization results using the IBL CBCT for various pediatric sites.^(^ ^16^ ^,^ ^17^ ^)^

Given the recent Image Gently Campaign, the increased risk of secondary malignancies for pediatric patients^(^ ^18^ ^)^ resulting from relatively low radiation doses, and the desire to increase the image quality of the IBL CBCT in order to further improve our localization capabilities, we have been studying an investigational imaging panel for use with the IBL. In this report, we detail the CNR and resolution measurements at various dose settings for the IBL CBCT with an investigational imaging panel.

## II. MATERIALS AND METHODS

Two Siemens Artiste linear accelerators with MVision were modified at our institution such that the MV‐CBCT would operate under an investigational IBL^(^ ^13^ ^)^ rather than the standard 6MV TBL. In addition, the standard imaging panel on one machine (AN‐9) was replaced with an investigational imager (AN‐19); the AN‐9 and AN‐19 are both from the PerkinElmer Flat Panel Detector series XRD 1640 (PerkinElmer, Waltham, MA). The major difference between the standard and investigational imaging panels is the scintillator screen. The scintillator on the investigational panel has a higher quantum efficiency and, therefore, should improve image quality. However, it costs approximately 50% more than the standard panel. The 1 mm copper plate, which sits just above the scintillator, may absorb a large fraction of the low‐energy photons produced by the IBL; therefore, the impact of its removal was studied.

The heterogeneity phantom used in this study was the CIRS electron density phantom model 02 (CIRS Inc., Norfolk, Virginia). This phantom can be used in the large diameter setting, which has an average diameter of 33 cm with two concentric rings of tissue‐equivalent inserts. The inner ring of inserts has a radius of 6.0 cm, and was used for this study. The phantom can also be used in a small diameter setting, with a phantom diameter of 18.0 cm and the same tissue insert positions. The various tissue‐equivalent inserts and their physical densities are listed in Table 1. After the machine modification, two CBCTs of the electron density phantom were acquired at each of the various “dose to isocenter” values using the IBL with the standard imager, the investigational imager with the copper panel, and the investigational imager without the copper panel. The reconstruction slice width was set to 5 mm for all images.

**Table 1:** A listing of the eight tissue‐equivalent inserts and physical densities of the electron density phantom.

*Low‐contrast Material*		*High‐contrast Material*	
*Tissue‐equivalent Inserts*	*Density Relative to Water*	*Tissue‐equivalent Inserts*	*Density Relative* to Water
Breast	−1%	Lung Inhale	−80%
Adipose	−3%	Lung Exhale	−50%
Muscle	+6%	Trabecular Bone	+16%
Liver	+7%	Dense Bone	+61%

**Figure 1:**
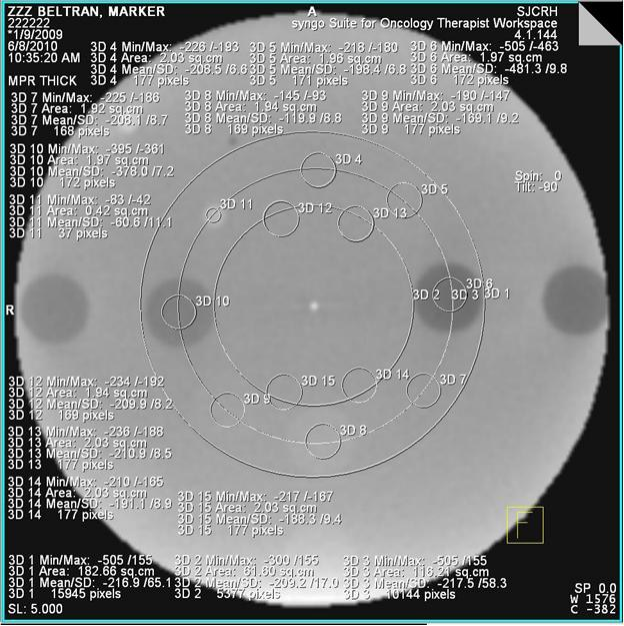
A 1.8 cGy at isocenter IBL CBCT image from the large phantom for the investigational panel without the copper plate. The region‐of‐interest circles for each insert and the nearest solid water are clearly visible.

For each CBCT, a region of interest (ROI) of approximately $2.0\;{\text{cm}}^{2}$ was outlined for each of the eight different electron density inserts in the inner ring of the phantom and for the water equivalent material near each insert. The ROI for the dense bone was smaller because of the insert size for that material. Figure 1 shows a 1.8 cGy at isocenter IBL CBCT image from the large phantom for the investigational panel without the copper plate. The regions of interest shown are the same as those used for all the CBCTs. The 3D module within the Siemens Coherence system was used to gather the data. This module allows one to draw ROIs, and gives pertinent statistics about that region. The mean pixel value (Signal) and standard deviation of the pixel values (Noise) for each insert were recorded. The contrast‐to‐noise ratio (CNR) for each tissue‐equivalent insert was calculated based on the following:

(1) $${\text{CNR}}_{\text{tissue}}=C_{\text{tissue}}/N_{\text{max}}$$

where $N_{\text{max}}=\text{Maximum}$ (Noise of Tissue equivalent or Noise of nearest Water equivalent), and $C_{\text{tissue}}=\text{absolute}$ value of [(Signal of Tissue equivalent) – (Signal of nearest Water equivalent)]. CNR results for the TBL with the standard imager and the CT used for simulation are included for completeness sake.^(^ ^15^ ^)^

Besides contrast, another important imaging property is resolution. Resolution measurements in line pairs per mm (lp/mm) of the CBCT for the various imaging panel setups were conducted. The standard image quality phantom provide by Siemens for routine CBCT quality assurance was used for these tests.

A qualitative clinical evaluation of the different imaging modalities was conducted. CBCT datasets from two patients (each of which were imaged twice with each panel configuration (total of 12 CBCTs)), were displayed for two physicians and three therapists without them knowing which dataset came from which imaging panel. They were asked to register the images to the treatment planning CT in the same manner as they do clinically. Then they were asked which CBCT set they felt was the best and the worst for registration. One patient had an intracranial target and the other an abdominal target. This test was used as a qualitative judge of practical image quality. Note, the dose to isocenter for each of these images was approximately 1 cGy. The use of the IBL on patients was conducted under an IRB‐approved study. Consent was obtained from both of the patients studied.

## III. RESULTS

**Figure 2:**
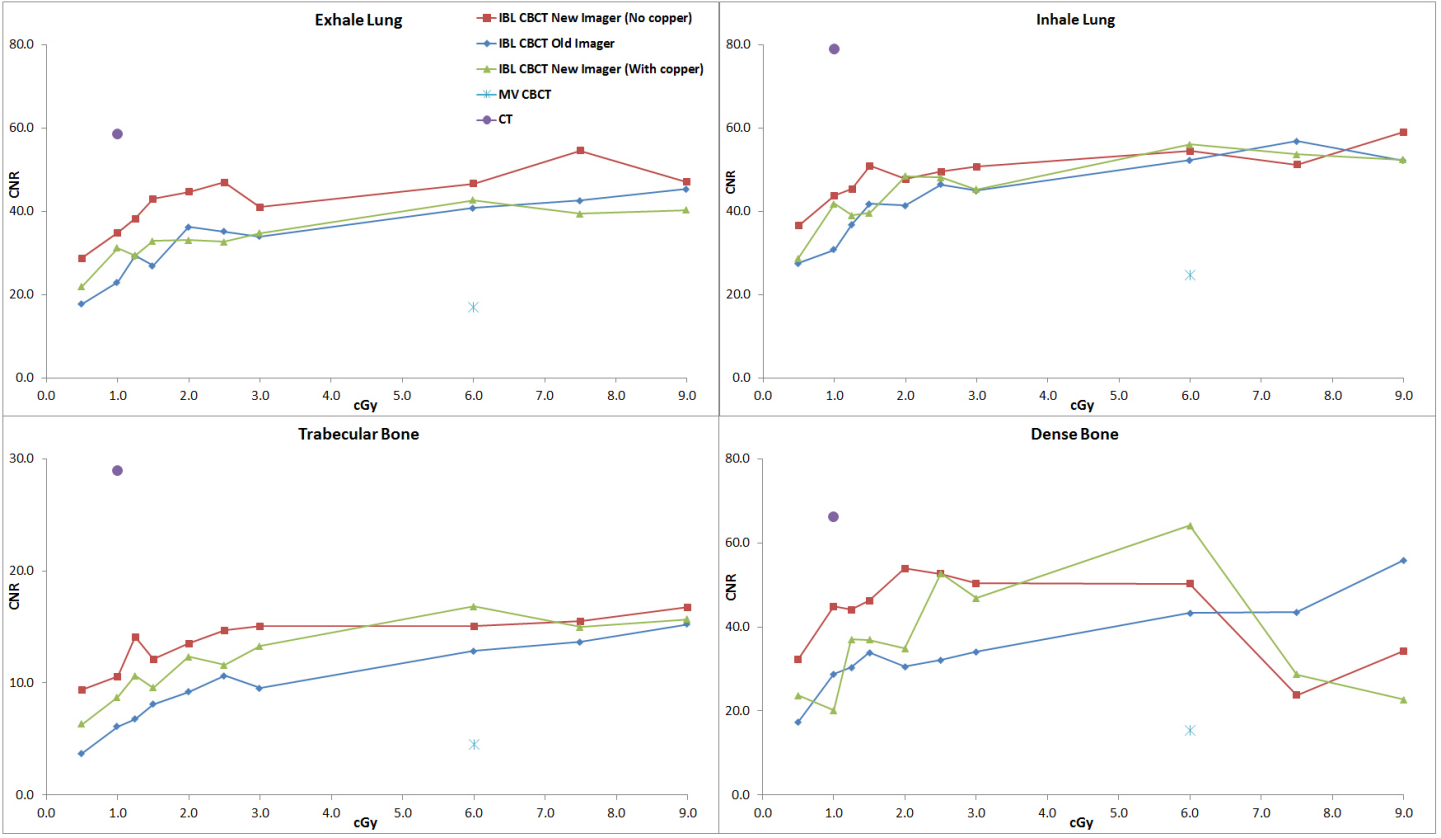
The CNR for the IBL with the various imaging panels as a function of dose for the high‐contrast material for the small phantom. Also plotted is the CNR for the CT scanner and for the TBL.

**Figure 3:**
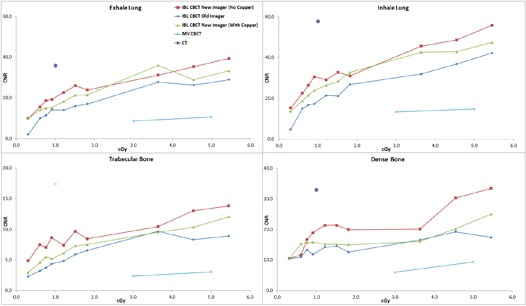
The CNR for the IBL with the various imaging panels as a function of dose for the high‐contrast material for the large phantom. Also plotted is the CNR for the CT scanner and the TBL.

**Figure 4:**
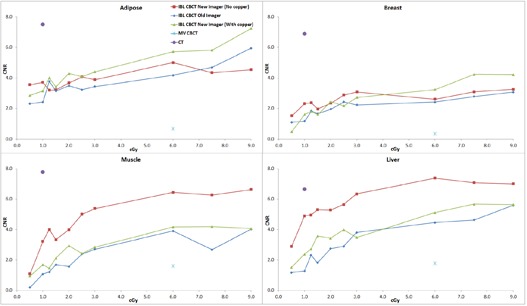
The CNR for the IBL with the various imaging panels as a function of dose for the low‐contrast material for the small phantom. Also plotted is the CNR for the CT scanner and the TBL.

**Figure 5:**
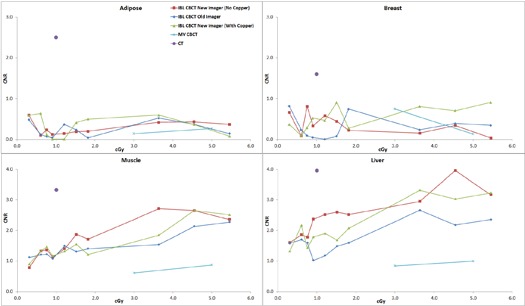
The CNR for the IBL with the various imaging panels as a function of dose for the low‐contrast material for the large phantom. Also plotted is the CNR for the CT scanner and the TBL.

The average of two measurements for the CNR of the muscle insert for the large phantom with 1.5 cGy at isocenter was 1.3 for the standard imager, 1.5 for the investigational imager with the copper plate, and 1.9 without the plate. Under the same conditions, the CNR of the trabecular bone insert was 5.9, 7.3, and 9.7, respectively. For the small phantom with the same dose to isocenter, the CNR for muscle was 1.7, 2.1, and 3.3, respectively. For the trabecular bone, the CNR was 8.1, 9.6, and 12.1. The average CNR of all 320 measurements (2 phantom sizes, 8 tissue inserts, 10 dose levels per phantom size, 2 CBCT sets each) for the investigational imager without the copper plate, with the copper plate, and for the standard imager was 16.1, 13.8, and 12.2, respectively. A paired one‐tailed t‐test of the investigational imager without copper and the standard imager gave p<0.00001, as did the investigational imager with the copper plate versus the standard imager. The investigational imager without the plate versus with the plate gave p=0.00001. All subgroup comparisons (e.g., the small phantom without the plate vs. the small phantom with the plate) reached statistical significance (p<0.05). Figure 2 displays the average CNR for the IBL with the various imaging panels as a function of dose for the high‐contrast material from the small phantom. Also plotted is the CNR for the CT scanner used for treatment simulation and the CNR for the TBL. Figure 3 is the average CNR for the high‐contrast material from the large phantom. Figures 4 and 5 are the average CNR as a function of dose for the low‐contrast material for the small and large phantom, respectively. Error bars are not shown on the figures; however, the average difference in CNR (with values > 6) between the two sets of measurements for the standard imager was 2.2%, 1.6% for the investigational imager with the copper plate, and 3.9% for the investigational imager without the plate. For small CNR values (< 6), the difference was always less than 0.5. Note on Fig. 2 that the CNR of the investigational imager for the dense bone drops at high doses. This may be caused by saturation in the imager.

The resolution at 1 cGy to isocenter was 0.37 lp/mm for the standard imager, 0.32 and 0.33 for the investigational imager with and without the copper plate, respectively. For the TBL with 4 cGy at isocenter, the resolution was 0.32 lp/mm.

The qualitative test ranked the CBCT of the investigational imager without the copper plate to be the best image, followed by the investigational imager with the copper plate and, lastly, the standard imager. All individuals ranked the images in this order, except one therapist ranked the investigational imager with the copper plate higher than without the plate for both sets of images from the intracranial patient. When asked what was better about the images, the overriding sentiment was that the investigational imager without the copper plate had better contrast, particularly for the abdominal area, which made it “easier to line up the anatomy”.

## IV. DISCUSSION & CONCLUSION

Based on the statistical analysis of the CNR measurements, the investigational imaging panel, with or without the copper plate, improves image quality as compared to the standard imager for IBL CBCTs, particularly in the low‐dose region (< 2 cGy). The investigational panel without the copper plate provides better CNR on average; however, there are some points in which the CNR is better for the investigational panel with the plate. When actual patient images were compared, the investigational imager without the copper plate was judged to produce the best IBL CBCT. With the investigational imager, an IBL CBCT with a dose of 2.5 cGy at isocenter may allow localize to soft tissue for some pediatric patients.

A comparison of image quality versus dose for other CBCT systems have not been conducted; however, several dosimetric comparisons have been undertaken. Gayou et al.,^(^ ^19^ ^)^ who measured CNR versus dose for the TBL CBCT, report a dose of approximately 9 cGy for clinical use. For the Elekta system, Islam et al.^(^ ^20^ ^)^ reported 3.0 cGy and 1.6 cGy at the center of a head and body phantom, respectively. Using the Varian OBI system, Song et al.^(^ ^21^ ^)^ reported 8.5 cGy and 4.1 cGy at the center of the head and body phantom, respectively, and they reported similar dose results as Islam et al. for the Elekta system. The CNR as a function of dose will likely always be higher for kV systems than either of the MV systems. However, as Ding et al.^(^ ^22^ ^)^ point out in their Monte Carlo dosimetric study of kV CBCT images in which they differentiated the dose to soft tissue and bones, the dose to bone can be 3 times higher than the dose to soft tissue. This is due to the predominant photoelectric effect of the kV photons in bone. This dose increase may be of consequence to a pediatric patient who has not yet reached growth maturity.

Examining the results of the resolution tests, it is clear that the standard imager has a higher resolution. However, clinically this slight degradation in resolution of the investigational imager is not a factor. When examining actual patient data, the CBCTs from the investigational imager were ranked higher than the standard imager. This is due to the better contrast and, therefore, overall better image quality. It should also be noted that this imaging system was optimized for the TBL with the standard imager. With specific optimization for the IBL and the investigational imager, further image quality gains may be realized.

## ACKNOWLEDGMENTS

Support provided by the American Lebanese Syrian Associated Charities (ALSAC) and Siemens OCS.
